# [6,13-Bis(2,4-dichloro­benzo­yl)-5,7,12,14-tetra­methyl­dibenzo[*b*,*i*][1,4,8,11]tetra­aza­cyclo­tetra­decinato- κ^4^ *N*]nickel(II) acetone monosolvate

**DOI:** 10.1107/S1600536812024671

**Published:** 2012-06-02

**Authors:** Bo-Kun Jiang, Xuan Shen, Xin Wang, Fan Su, Dun-Ru Zhu

**Affiliations:** a State Key Laboratory of Materials-Oriented Chemical Engineering, College of Chemistry and Chemical Engineering, Nanjing University of Technology, Nanjing 210009, People’s Republic of China

## Abstract

In the title complex, [Ni(C_36_H_26_Cl_4_N_4_O_2_)]·C_3_H_6_O, two 2,4-dichloro­benzoyl groups are grafted onto the methine groups of the Ni^II^ complex Ni(tmtaa) (H_2_tmtaa = 5,7,12,14-tetra­methyl-4,11-dihydro­dibenzo[*b*,*i*][1,4,8,11]tetra­aza­cyclo­tetra­decine). The complex has the shape of a saddle. The Ni atom is tetra­coordinated by the four N atoms of the macrocycle, forming a slightly tetra­hedrally distorted square-planar geometry. The metal is displaced by 0.0101 (8) Å from the N_4_ mean plane. The aromatic rings of the 2,4-dichloro­benzoyl groups form dihedral angles of 87.1 (2) and 82.1 (2)° with the N_4_ mean plane

## Related literature

For general background to the chemistry of H_2_tmtaa and its complexes, see: Jäger (1969 ▶); Cotton & Czuchajowska (1990 ▶); Mountford (1998 ▶). For the syntheses and structures of related compounds, see: Sakata *et al.* (1996 ▶); Eilmes *et al.* (2001 ▶); Shen *et al.* (2008 ▶).
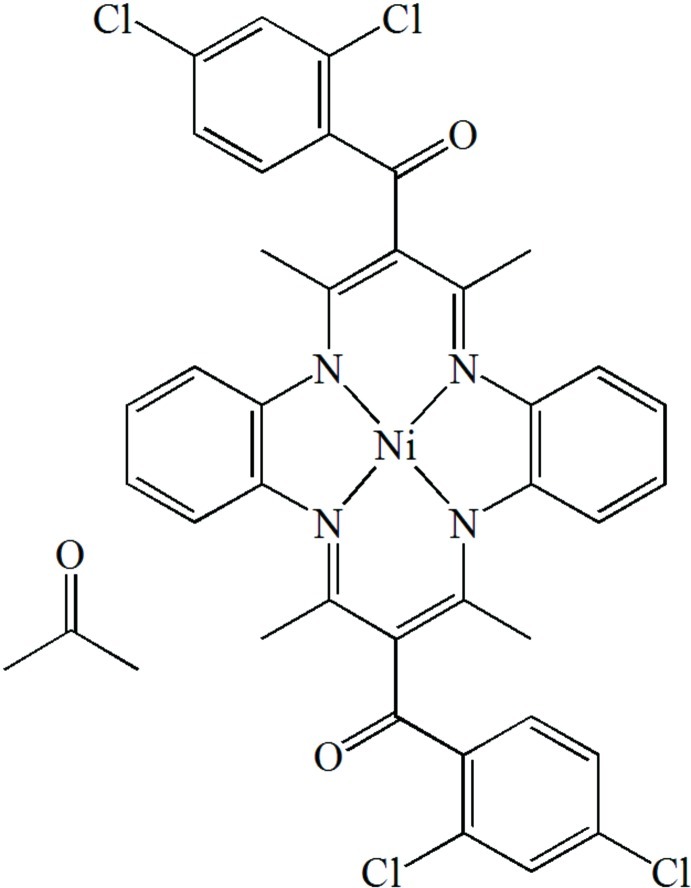


## Experimental

### 

#### Crystal data

[Ni(C_36_H_26_Cl_4_N_4_O_2_)]·C_3_H_6_O
*M* *_r_* = 805.20
Monoclinic, 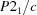

*a* = 11.546 (3) Å
*b* = 27.134 (7) Å
*c* = 12.149 (3) Å
β = 110.372 (4)°
*V* = 3568.1 (17) Å^3^
*Z* = 4
Mo *K*α radiation
μ = 0.89 mm^−1^
*T* = 296 K
0.12 × 0.08 × 0.06 mm

#### Data collection

Bruker APEXII CCD diffractometer
Absorption correction: multi-scan (*SADABS*; Sheldrick, 1996 ▶) *T*_min_ = 0.901, *T*_max_ = 0.949
21315 measured reflections
6137 independent reflections
3204 reflections with *I* > 2σ(*I*)
*R*_int_ = 0.172

#### Refinement

*R*[*F* ^2^ > 2σ(*F* ^2^)] = 0.078
*wR*(*F* ^2^) = 0.190
*S* = 1.04
6137 reflections
460 parameters
H-atom parameters constrained
Δρ_max_ = 1.65 e Å^−3^
Δρ_min_ = −2.37 e Å^−3^

### 

Data collection: *APEX2* (Bruker, 2005 ▶); cell refinement: *SAINT* (Bruker, 2005 ▶); data reduction: *SAINT*; program(s) used to solve structure: *SHELXS97* (Sheldrick, 2008 ▶); program(s) used to refine structure: *SHELXL97* (Sheldrick, 2008 ▶); molecular graphics: *SHELXTL* (Sheldrick, 2008 ▶); software used to prepare material for publication: *SHELXTL*.

## Supplementary Material

Crystal structure: contains datablock(s) I, global. DOI: 10.1107/S1600536812024671/rz2759sup1.cif

Structure factors: contains datablock(s) I. DOI: 10.1107/S1600536812024671/rz2759Isup2.hkl

Supplementary material file. DOI: 10.1107/S1600536812024671/rz2759Isup3.mol

Additional supplementary materials:  crystallographic information; 3D view; checkCIF report

## Figures and Tables

**Fig. 1.**
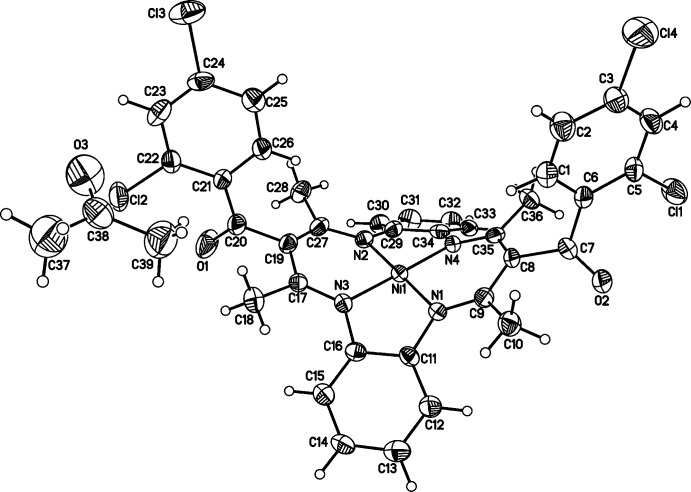
The molecular structure of the title compound, showing 30% probability displacement ellipsoids.

**Fig. 2.**
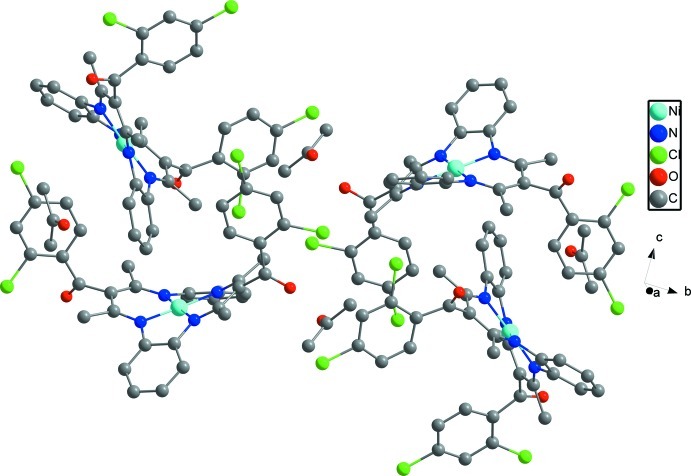
A view of the unit cell packing along the a axis. H-atoms have been omitted for clarity.

## supplementary crystallographic information

### Comment

H_2_tmtaa, a versatile ligand for transition and main group metals, is a macrocyclic compound with a 14-membered ring and has a structure and properties similar to porphyrin and phthalocyanine. The distinctive individual characteristics of this synthetic macrocycle make it interesting in a wide range of chemical areas (Cotton *et al.*, 1990; Mountford, 1998). The syntheses of modified free H_2_tmtaa or tmtaa complexes through substitution at the *γ* and *γ'* positions have been extensively researched (Sakata *et al.*, 1996; Eilmes *et al.*, 2001; Shen *et al.*, 2008). As a continuation of the investigation on the reactivity of *γ* and *γ'* positions in Ni(tmtaa) (Jäger, 1969), we herein report the synthesis and crystal structure of the title compound.

The molecular structure of the title compound is illustrated in Fig. 1. The non-planar saddle-shaped conformation of the Nitmtaa is maintained with two 2,4-dichlorobenzoyl groups folding towards the central metal. The dihedral angle between the benzene rings of the tmtaa ligand is 62.4 (2)°. The Ni atom is coordinated to four N atoms of tmtaa in a sligthly tetrahedrally distorted coordination geometry and protrudes from the N_4_ plane by only 0.0101 (8) Å. The dihedral angle between two aromatic rings in the grafted substituents is 15.98° and both of them are almost perpendicular to the N_4_ plane forming dihedral angles of 87.1 (2) and 82.1 (2)°, respectively. The Ni–N bond distances range from 1.857 (4) to 1.862 (4) Å with a mean value of 1.860 (4) Å. In the crystal structure (Fig. 2), no hydrogen bonds or other weak intermolecular interactions are observed.

### Experimental

After a solution of Ni(tmtaa) (0.602 g, 1.50 mmol) and triethylamine (2 ml) in dry toluene (50 ml) was stirred at room temperature under nitrogen atmosphere for 10 min, a solution of 2,4-dichlorobenzoyl chloride (0.670 g, 3.20 mmol) in dry toluene (50 ml) was slowly added dropwise and the reaction mixture was further stirred at 80°C for 12 h. After been cooled to room temperature, the reacted mixture was filtered to eliminate the triethylamine hydrochloride formed. Evaporation of the filtrate resulted in a dark green powder. The powder was purified and separated on an alumina chromatographic column using petroleum ether and ethyl acetate (8:1 *v*/*v*) as the eluant. The product was recrystallized from acetone to give dark green crystals of the title compound in a yield of 0.652 g (54%).

### Refinement

All H atoms were positioned geometrically and refined using a riding model with C—H = 0.93 and 0.96 Å for aryl and methyl H–atoms, respectively. The *U*_iso_(H) were allowed at 1.2*U*_eq_(C) or 1.5*U*_eq_(C) for methyl H atoms.

### Crystal data

**Table:**

[Ni(C_36_H_26_Cl_4_N_4_O_2_)]·C_3_H_6_O	*F*(000) = 1656
*M**_r_* = 805.20	*D*_x_ = 1.499 Mg m^−^^3^
Monoclinic, *P*2_1_/*c*	Mo *K*α radiation, λ = 0.71073 Å
Hall symbol: -P 2ybc	Cell parameters from 34 reflections
*a* = 11.546 (3) Å	θ = 2.3–26.2°
*b* = 27.134 (7) Å	µ = 0.89 mm^−^^1^
*c* = 12.149 (3) Å	*T* = 296 K
β = 110.372 (4)°	Block, dark green
*V* = 3568.1 (17) Å^3^	0.12 × 0.08 × 0.06 mm
*Z* = 4	

### Data collection

**Table:**

Bruker APEXII CCD diffractometer	6137 independent reflections
Radiation source: fine-focus sealed tube	3204 reflections with *I* > 2σ(*I*)
Graphite monochromator	*R*_int_ = 0.172
ω scans	θ_max_ = 25.0°, θ_min_ = 1.9°
Absorption correction: multi-scan (*SADABS*; Sheldrick, 1996)	*h* = −13→13
*T*_min_ = 0.901, *T*_max_ = 0.949	*k* = −32→32
21315 measured reflections	*l* = −14→13

### Refinement

**Table:**

Refinement on *F*^2^	Primary atom site location: structure-invariant direct methods
Least-squares matrix: full	Secondary atom site location: difference Fourier map
*R*[*F*^2^ > 2σ(*F*^2^)] = 0.078	Hydrogen site location: inferred from neighbouring sites
*wR*(*F*^2^) = 0.190	H-atom parameters constrained
*S* = 1.04	*w* = 1/[σ^2^(*F*_o_^2^) + (0.067*P*)^2^] where *P* = (*F*_o_^2^ + 2*F*_c_^2^)/3
6137 reflections	(Δ/σ)_max_ = 0.002
460 parameters	Δρ_max_ = 1.65 e Å^−^^3^
0 restraints	Δρ_min_ = −2.37 e Å^−^^3^

### Special details

**Table:**

Geometry. All e.s.d.'s (except the e.s.d. in the dihedral angle between two l.s. planes) are estimated using the full covariance matrix. The cell e.s.d.'s are taken into account individually in the estimation of e.s.d.'s in distances, angles and torsion angles; correlations between e.s.d.'s in cell parameters are only used when they are defined by crystal symmetry. An approximate (isotropic) treatment of cell e.s.d.'s is used for estimating e.s.d.'s involving l.s. planes.
Refinement. Refinement of *F*^2^ against ALL reflections. The weighted *R*-factor *wR* and goodness of fit *S* are based on *F*^2^, conventional *R*-factors *R* are based on *F*, with *F* set to zero for negative *F*^2^. The threshold expression of *F*^2^ > σ(*F*^2^) is used only for calculating *R*-factors(gt) *etc*. and is not relevant to the choice of reflections for refinement. *R*-factors based on *F*^2^ are statistically about twice as large as those based on *F*, and *R*- factors based on ALL data will be even larger.

### Fractional atomic coordinates and isotropic or equivalent isotropic displacement parameters (Å^2^)

**Table:**

	*x*	*y*	*z*	*U*_iso_*/*U*_eq_	
Ni1	2.16249 (6)	−0.17047 (3)	2.42062 (6)	0.0368 (2)	
N1	2.3256 (4)	−0.17941 (19)	2.4315 (4)	0.0391 (12)	
N2	1.9992 (4)	−0.16079 (19)	2.4095 (4)	0.0417 (13)	
N3	2.1638 (4)	−0.11609 (19)	2.3281 (4)	0.0401 (12)	
N4	2.1601 (4)	−0.2260 (2)	2.5097 (4)	0.0388 (12)	
Cl1	2.4561 (2)	−0.41867 (9)	2.59903 (17)	0.0823 (6)	
Cl2	1.6819 (2)	−0.03283 (9)	1.90340 (18)	0.0834 (7)	
Cl3	1.60697 (19)	−0.21256 (10)	1.71613 (17)	0.0919 (8)	
Cl4	2.2994 (2)	−0.47349 (11)	2.1476 (2)	0.0997 (9)	
O1	1.8035 (6)	−0.0458 (3)	2.1541 (5)	0.104 (2)	
O2	2.5123 (5)	−0.3123 (2)	2.5822 (6)	0.0883 (18)	
O3	1.8191 (6)	−0.0670 (3)	1.6827 (7)	0.122 (3)	
C1	2.3202 (6)	−0.3429 (3)	2.2984 (7)	0.066 (2)	
H1A	2.2980	−0.3107	2.2740	0.079*	
C2	2.2992 (6)	−0.3796 (3)	2.2159 (7)	0.067 (2)	
H2A	2.2675	−0.3721	2.1363	0.081*	
C3	2.3254 (6)	−0.4273 (3)	2.2519 (6)	0.059 (2)	
C4	2.3716 (6)	−0.4398 (3)	2.3686 (7)	0.062 (2)	
H4A	2.3846	−0.4726	2.3917	0.074*	
C5	2.3982 (5)	−0.4026 (3)	2.4505 (6)	0.0518 (18)	
C6	2.3747 (5)	−0.3530 (3)	2.4192 (5)	0.0477 (16)	
C7	2.4127 (6)	−0.3101 (3)	2.5037 (6)	0.0534 (17)	
C8	2.3334 (5)	−0.2649 (3)	2.4819 (5)	0.0451 (15)	
C9	2.3812 (5)	−0.2225 (3)	2.4453 (5)	0.0435 (16)	
C10	2.4962 (5)	−0.2289 (3)	2.4109 (6)	0.0584 (19)	
H10A	2.5193	−0.1977	2.3878	0.088*	
H10B	2.4782	−0.2517	2.3465	0.088*	
H10C	2.5629	−0.2416	2.4767	0.088*	
C11	2.3762 (5)	−0.1343 (2)	2.4110 (5)	0.0430 (16)	
C12	2.5004 (5)	−0.1204 (3)	2.4554 (6)	0.0565 (19)	
H12A	2.5599	−0.1432	2.4963	0.068*	
C13	2.5356 (6)	−0.0732 (3)	2.4393 (6)	0.066 (2)	
H13A	2.6186	−0.0643	2.4678	0.079*	
C14	2.4468 (6)	−0.0391 (3)	2.3804 (6)	0.0574 (19)	
H14A	2.4703	−0.0074	2.3678	0.069*	
C15	2.3229 (6)	−0.0518 (3)	2.3403 (5)	0.0517 (17)	
H15A	2.2636	−0.0283	2.3035	0.062*	
C16	2.2870 (5)	−0.0986 (2)	2.3540 (5)	0.0433 (16)	
C17	2.0664 (5)	−0.0984 (2)	2.2450 (5)	0.0425 (15)	
C18	2.0783 (6)	−0.0667 (3)	2.1465 (6)	0.061 (2)	
H18A	2.1642	−0.0612	2.1591	0.092*	
H18B	2.0381	−0.0356	2.1453	0.092*	
H18C	2.0403	−0.0832	2.0727	0.092*	
C19	1.9455 (5)	−0.1101 (3)	2.2400 (5)	0.0486 (17)	
C20	1.8430 (6)	−0.0871 (3)	2.1423 (6)	0.0563 (18)	
C21	1.7900 (5)	−0.1152 (3)	2.0300 (5)	0.0479 (17)	
C22	1.7121 (5)	−0.0950 (3)	1.9237 (6)	0.0519 (18)	
C23	1.6576 (6)	−0.1247 (4)	1.8269 (6)	0.067 (2)	
H23A	1.6057	−0.1111	1.7569	0.081*	
C24	1.6808 (6)	−0.1743 (3)	1.8351 (6)	0.058 (2)	
C25	1.7608 (6)	−0.1952 (3)	1.9366 (6)	0.063 (2)	
H25A	1.7788	−0.2287	1.9403	0.076*	
C26	1.8134 (6)	−0.1652 (3)	2.0320 (6)	0.0557 (18)	
H26A	1.8670	−0.1792	2.1008	0.067*	
C27	1.9141 (5)	−0.1369 (3)	2.3232 (5)	0.0501 (18)	
C28	1.7787 (5)	−0.1420 (3)	2.3064 (6)	0.066 (2)	
H28A	1.7700	−0.1612	2.3695	0.099*	
H28B	1.7366	−0.1582	2.2330	0.099*	
H28C	1.7436	−0.1099	2.3061	0.099*	
C29	1.9799 (5)	−0.1845 (3)	2.5064 (5)	0.0453 (16)	
C30	1.8935 (5)	−0.1707 (3)	2.5579 (6)	0.057 (2)	
H30A	1.8356	−0.1463	2.5236	0.069*	
C31	1.8948 (7)	−0.1937 (3)	2.6600 (7)	0.072 (2)	
H31A	1.8358	−0.1850	2.6927	0.087*	
C32	1.9796 (6)	−0.2283 (3)	2.7133 (6)	0.066 (2)	
H32A	1.9785	−0.2431	2.7820	0.080*	
C33	2.0680 (5)	−0.2418 (3)	2.6664 (5)	0.0507 (17)	
H33A	2.1275	−0.2653	2.7037	0.061*	
C34	2.0669 (5)	−0.2199 (3)	2.5620 (5)	0.0451 (16)	
C35	2.2295 (5)	−0.2652 (3)	2.5173 (5)	0.0472 (17)	
C36	2.1961 (6)	−0.3143 (3)	2.5607 (7)	0.064 (2)	
H36A	2.1242	−0.3099	2.5823	0.096*	
H36B	2.2639	−0.3252	2.6278	0.096*	
H36C	2.1792	−0.3384	2.4994	0.096*	
C37	1.9714 (9)	−0.0368 (5)	1.6169 (9)	0.133 (5)	
H37A	1.9020	−0.0267	1.5501	0.200*	
H37B	2.0215	−0.0594	1.5922	0.200*	
H37C	2.0196	−0.0084	1.6523	0.200*	
C38	1.9267 (8)	−0.0612 (3)	1.7031 (8)	0.077 (2)	
C39	2.0198 (9)	−0.0793 (5)	1.8118 (9)	0.113 (4)	
H39A	1.9794	−0.0943	1.8602	0.170*	
H39B	2.0696	−0.0522	1.8532	0.170*	
H39C	2.0714	−0.1032	1.7931	0.170*	

### Atomic displacement parameters (Å^2^)

**Table:**

	*U*^11^	*U*^22^	*U*^33^	*U*^12^	*U*^13^	*U*^23^
Ni1	0.0381 (3)	0.0335 (5)	0.0383 (4)	0.0003 (3)	0.0126 (3)	0.0001 (4)
N1	0.040 (2)	0.034 (4)	0.042 (3)	−0.001 (2)	0.013 (2)	−0.001 (2)
N2	0.039 (2)	0.045 (4)	0.042 (3)	−0.001 (2)	0.015 (2)	−0.002 (2)
N3	0.046 (2)	0.037 (4)	0.040 (3)	0.001 (2)	0.016 (2)	0.002 (2)
N4	0.039 (2)	0.035 (4)	0.039 (3)	0.000 (2)	0.009 (2)	0.003 (2)
Cl1	0.1151 (15)	0.0569 (17)	0.0594 (12)	−0.0017 (13)	0.0108 (11)	0.0115 (11)
Cl2	0.0993 (14)	0.0670 (18)	0.0752 (14)	0.0204 (13)	0.0193 (11)	0.0261 (12)
Cl3	0.1011 (14)	0.119 (2)	0.0605 (12)	−0.0510 (15)	0.0348 (11)	−0.0259 (12)
Cl4	0.0996 (14)	0.109 (2)	0.0915 (16)	0.0098 (14)	0.0340 (13)	−0.0483 (15)
O1	0.109 (4)	0.080 (6)	0.085 (4)	0.044 (4)	−0.016 (3)	−0.019 (4)
O2	0.070 (3)	0.048 (4)	0.112 (5)	0.014 (3)	−0.012 (3)	−0.013 (4)
O3	0.090 (4)	0.130 (8)	0.160 (7)	0.022 (4)	0.060 (5)	0.014 (5)
C1	0.064 (4)	0.055 (6)	0.068 (5)	0.011 (4)	0.011 (4)	0.004 (4)
C2	0.077 (5)	0.066 (7)	0.060 (5)	0.011 (4)	0.025 (4)	0.003 (4)
C3	0.059 (4)	0.057 (6)	0.064 (5)	0.008 (4)	0.027 (4)	−0.012 (4)
C4	0.065 (4)	0.045 (5)	0.080 (5)	0.009 (4)	0.030 (4)	−0.009 (4)
C5	0.054 (3)	0.047 (5)	0.052 (4)	0.013 (3)	0.014 (3)	0.010 (4)
C6	0.043 (3)	0.044 (5)	0.053 (4)	0.009 (3)	0.012 (3)	0.000 (3)
C7	0.059 (4)	0.035 (5)	0.062 (4)	0.003 (3)	0.015 (4)	0.004 (4)
C8	0.042 (3)	0.033 (5)	0.053 (4)	0.009 (3)	0.008 (3)	0.000 (3)
C9	0.044 (3)	0.043 (5)	0.043 (3)	0.003 (3)	0.013 (3)	−0.005 (3)
C10	0.055 (3)	0.054 (5)	0.070 (4)	0.007 (3)	0.026 (3)	−0.001 (4)
C11	0.048 (3)	0.034 (4)	0.052 (4)	0.001 (3)	0.023 (3)	−0.002 (3)
C12	0.049 (3)	0.054 (5)	0.068 (4)	−0.002 (3)	0.022 (3)	0.006 (4)
C13	0.059 (4)	0.067 (7)	0.072 (5)	−0.015 (4)	0.022 (4)	−0.004 (4)
C14	0.061 (4)	0.044 (5)	0.070 (5)	−0.014 (4)	0.025 (4)	0.002 (4)
C15	0.060 (3)	0.046 (5)	0.051 (4)	−0.002 (3)	0.021 (3)	0.001 (3)
C16	0.045 (3)	0.044 (5)	0.044 (3)	−0.005 (3)	0.020 (3)	−0.003 (3)
C17	0.051 (3)	0.036 (5)	0.036 (3)	0.001 (3)	0.009 (3)	−0.006 (3)
C18	0.070 (4)	0.056 (6)	0.057 (4)	0.008 (4)	0.022 (4)	0.012 (4)
C19	0.043 (3)	0.048 (5)	0.049 (4)	0.008 (3)	0.008 (3)	−0.004 (3)
C20	0.055 (4)	0.047 (5)	0.058 (4)	0.011 (3)	0.008 (3)	−0.001 (4)
C21	0.040 (3)	0.049 (5)	0.049 (4)	0.002 (3)	0.007 (3)	0.006 (3)
C22	0.048 (3)	0.054 (5)	0.055 (4)	0.004 (3)	0.019 (3)	0.012 (4)
C23	0.047 (3)	0.107 (8)	0.043 (4)	−0.006 (4)	0.010 (3)	0.010 (5)
C24	0.056 (4)	0.071 (7)	0.048 (4)	−0.029 (4)	0.019 (3)	−0.010 (4)
C25	0.066 (4)	0.056 (6)	0.068 (5)	0.002 (4)	0.024 (4)	0.001 (4)
C26	0.064 (4)	0.043 (5)	0.053 (4)	0.007 (3)	0.011 (3)	0.001 (4)
C27	0.038 (3)	0.057 (6)	0.049 (4)	0.004 (3)	0.008 (3)	−0.008 (3)
C28	0.042 (3)	0.081 (7)	0.071 (5)	0.008 (4)	0.015 (3)	0.004 (4)
C29	0.038 (3)	0.049 (5)	0.047 (3)	−0.002 (3)	0.013 (3)	−0.002 (3)
C30	0.047 (3)	0.066 (6)	0.064 (4)	−0.002 (3)	0.025 (3)	−0.001 (4)
C31	0.071 (4)	0.088 (7)	0.076 (5)	−0.006 (5)	0.049 (4)	0.002 (5)
C32	0.075 (4)	0.075 (7)	0.060 (4)	−0.009 (4)	0.037 (4)	0.006 (4)
C33	0.055 (3)	0.045 (5)	0.046 (4)	−0.011 (3)	0.010 (3)	0.007 (3)
C34	0.046 (3)	0.045 (5)	0.042 (3)	−0.017 (3)	0.014 (3)	−0.005 (3)
C35	0.041 (3)	0.047 (5)	0.043 (3)	−0.011 (3)	0.001 (3)	0.001 (3)
C36	0.066 (4)	0.039 (5)	0.086 (5)	−0.004 (4)	0.024 (4)	0.005 (4)
C37	0.112 (7)	0.172 (15)	0.116 (9)	−0.021 (8)	0.039 (7)	0.033 (9)
C38	0.087 (5)	0.058 (7)	0.093 (6)	0.008 (5)	0.039 (5)	−0.002 (5)
C39	0.123 (7)	0.117 (12)	0.088 (7)	0.009 (7)	0.021 (6)	0.003 (7)

### Geometric parameters (Å, º)

**Table:**

Ni1—N1	1.857 (4)	C15—H15A	0.9300
Ni1—N3	1.858 (5)	C17—C19	1.413 (7)
Ni1—N4	1.861 (5)	C17—C18	1.519 (8)
Ni1—N2	1.862 (4)	C18—H18A	0.9600
N1—C9	1.315 (8)	C18—H18B	0.9600
N1—C11	1.415 (7)	C18—H18C	0.9600
N2—C27	1.330 (8)	C19—C27	1.391 (9)
N2—C29	1.425 (7)	C19—C20	1.490 (9)
N3—C17	1.313 (7)	C20—C21	1.495 (9)
N3—C16	1.426 (7)	C21—C26	1.383 (10)
N4—C35	1.316 (8)	C21—C22	1.403 (9)
N4—C34	1.437 (6)	C22—C23	1.383 (10)
Cl1—C5	1.747 (7)	C23—C24	1.371 (11)
Cl2—C22	1.723 (8)	C23—H23A	0.9300
Cl3—C24	1.741 (7)	C24—C25	1.379 (10)
Cl4—C3	1.732 (7)	C25—C26	1.372 (10)
O1—C20	1.238 (9)	C25—H25A	0.9300
O2—C7	1.213 (8)	C26—H26A	0.9300
O3—C38	1.190 (9)	C27—C28	1.511 (7)
C1—C2	1.374 (11)	C28—H28A	0.9600
C1—C6	1.407 (9)	C28—H28B	0.9600
C1—H1A	0.9300	C28—H28C	0.9600
C2—C3	1.365 (11)	C29—C34	1.384 (9)
C2—H2A	0.9300	C29—C30	1.400 (7)
C3—C4	1.372 (10)	C30—C31	1.384 (10)
C4—C5	1.376 (10)	C30—H30A	0.9300
C4—H4A	0.9300	C31—C32	1.348 (11)
C5—C6	1.399 (10)	C31—H31A	0.9300
C6—C7	1.511 (10)	C32—C33	1.382 (8)
C7—C8	1.498 (9)	C32—H32A	0.9300
C8—C35	1.409 (7)	C33—C34	1.397 (8)
C8—C9	1.414 (9)	C33—H33A	0.9300
C9—C10	1.535 (6)	C35—C36	1.530 (9)
C10—H10A	0.9600	C36—H36A	0.9600
C10—H10B	0.9600	C36—H36B	0.9600
C10—H10C	0.9600	C36—H36C	0.9600
C11—C12	1.397 (8)	C37—C38	1.475 (11)
C11—C16	1.408 (8)	C37—H37A	0.9600
C12—C13	1.378 (10)	C37—H37B	0.9600
C12—H12A	0.9300	C37—H37C	0.9600
C13—C14	1.381 (10)	C38—C39	1.467 (13)
C13—H13A	0.9300	C39—H39A	0.9600
C14—C15	1.385 (8)	C39—H39B	0.9600
C14—H14A	0.9300	C39—H39C	0.9600
C15—C16	1.364 (9)		
			
N1—Ni1—N3	85.7 (2)	H18B—C18—H18C	109.5
N1—Ni1—N4	94.2 (2)	C27—C19—C17	126.1 (6)
N3—Ni1—N4	178.5 (2)	C27—C19—C20	117.5 (5)
N1—Ni1—N2	179.4 (2)	C17—C19—C20	116.1 (6)
N3—Ni1—N2	93.8 (2)	O1—C20—C19	120.7 (7)
N4—Ni1—N2	86.4 (2)	O1—C20—C21	121.1 (6)
C9—N1—C11	125.3 (4)	C19—C20—C21	118.3 (6)
C9—N1—Ni1	124.2 (4)	C26—C21—C22	117.3 (7)
C11—N1—Ni1	110.2 (4)	C26—C21—C20	117.9 (6)
C27—N2—C29	125.9 (4)	C22—C21—C20	124.7 (7)
C27—N2—Ni1	125.2 (3)	C23—C22—C21	120.7 (8)
C29—N2—Ni1	108.8 (4)	C23—C22—Cl2	116.1 (6)
C17—N3—C16	124.7 (5)	C21—C22—Cl2	123.2 (6)
C17—N3—Ni1	124.7 (4)	C24—C23—C22	119.5 (7)
C16—N3—Ni1	110.4 (4)	C24—C23—H23A	120.3
C35—N4—C34	126.5 (5)	C22—C23—H23A	120.3
C35—N4—Ni1	124.3 (3)	C23—C24—C25	121.4 (7)
C34—N4—Ni1	109.2 (4)	C23—C24—Cl3	119.9 (6)
C2—C1—C6	121.3 (8)	C25—C24—Cl3	118.7 (7)
C2—C1—H1A	119.3	C26—C25—C24	118.3 (8)
C6—C1—H1A	119.3	C26—C25—H25A	120.8
C3—C2—C1	119.4 (7)	C24—C25—H25A	120.8
C3—C2—H2A	120.3	C25—C26—C21	122.7 (7)
C1—C2—H2A	120.3	C25—C26—H26A	118.7
C2—C3—C4	121.9 (7)	C21—C26—H26A	118.7
C2—C3—Cl4	119.3 (6)	N2—C27—C19	121.4 (5)
C4—C3—Cl4	118.8 (6)	N2—C27—C28	120.4 (6)
C3—C4—C5	118.3 (7)	C19—C27—C28	118.0 (6)
C3—C4—H4A	120.8	C27—C28—H28A	109.5
C5—C4—H4A	120.8	C27—C28—H28B	109.5
C4—C5—C6	122.4 (6)	H28A—C28—H28B	109.5
C4—C5—Cl1	118.2 (6)	C27—C28—H28C	109.5
C6—C5—Cl1	119.3 (5)	H28A—C28—H28C	109.5
C5—C6—C1	116.4 (7)	H28B—C28—H28C	109.5
C5—C6—C7	124.9 (6)	C34—C29—C30	118.5 (6)
C1—C6—C7	118.5 (7)	C34—C29—N2	114.8 (4)
O2—C7—C8	122.2 (7)	C30—C29—N2	126.1 (6)
O2—C7—C6	117.9 (6)	C31—C30—C29	119.5 (7)
C8—C7—C6	119.7 (6)	C31—C30—H30A	120.2
C35—C8—C9	124.5 (6)	C29—C30—H30A	120.2
C35—C8—C7	118.3 (6)	C32—C31—C30	121.6 (5)
C9—C8—C7	116.2 (4)	C32—C31—H31A	119.2
N1—C9—C8	122.5 (4)	C30—C31—H31A	119.2
N1—C9—C10	119.8 (6)	C31—C32—C33	120.3 (6)
C8—C9—C10	117.5 (6)	C31—C32—H32A	119.9
C9—C10—H10A	109.5	C33—C32—H32A	119.9
C9—C10—H10B	109.5	C32—C33—C34	119.1 (7)
H10A—C10—H10B	109.5	C32—C33—H33A	120.4
C9—C10—H10C	109.5	C34—C33—H33A	120.4
H10A—C10—H10C	109.5	C29—C34—C33	121.0 (5)
H10B—C10—H10C	109.5	C29—C34—N4	113.2 (5)
C12—C11—C16	118.7 (6)	C33—C34—N4	125.4 (6)
C12—C11—N1	126.7 (6)	N4—C35—C8	122.5 (6)
C16—C11—N1	113.9 (5)	N4—C35—C36	120.5 (5)
C13—C12—C11	120.7 (7)	C8—C35—C36	117.0 (6)
C13—C12—H12A	119.6	C35—C36—H36A	109.5
C11—C12—H12A	119.6	C35—C36—H36B	109.5
C12—C13—C14	119.6 (6)	H36A—C36—H36B	109.5
C12—C13—H13A	120.2	C35—C36—H36C	109.5
C14—C13—H13A	120.2	H36A—C36—H36C	109.5
C13—C14—C15	120.3 (7)	H36B—C36—H36C	109.5
C13—C14—H14A	119.9	C38—C37—H37A	109.5
C15—C14—H14A	119.9	C38—C37—H37B	109.5
C16—C15—C14	120.7 (7)	H37A—C37—H37B	109.5
C16—C15—H15A	119.6	C38—C37—H37C	109.5
C14—C15—H15A	119.6	H37A—C37—H37C	109.5
C15—C16—C11	119.9 (5)	H37B—C37—H37C	109.5
C15—C16—N3	127.2 (6)	O3—C38—C39	121.8 (8)
C11—C16—N3	112.5 (5)	O3—C38—C37	120.7 (9)
N3—C17—C19	121.4 (5)	C39—C38—C37	117.5 (8)
N3—C17—C18	121.6 (5)	C38—C39—H39A	109.5
C19—C17—C18	116.9 (6)	C38—C39—H39B	109.5
C17—C18—H18A	109.5	H39A—C39—H39B	109.5
C17—C18—H18B	109.5	C38—C39—H39C	109.5
H18A—C18—H18B	109.5	H39A—C39—H39C	109.5
C17—C18—H18C	109.5	H39B—C39—H39C	109.5
H18A—C18—H18C	109.5		
